# Vitamin K2 Induces Mitochondria-Related Apoptosis in Human Bladder Cancer Cells via ROS and JNK/p38 MAPK Signal Pathways

**DOI:** 10.1371/journal.pone.0161886

**Published:** 2016-08-29

**Authors:** Fengsen Duan, Yuejin Yu, Rijian Guan, Zhiliang Xu, Huageng Liang, Ling Hong

**Affiliations:** 1 Department of Genetics and Developmental Biology, College of Life Science and Technology, Huazhong University of Science and Technology, Wuhan, Hubei, P.R. China; 2 Department of Urology, Union Hospital, Tongji Medical College, Huazhong University of Science and Technology, Wuhan, Hubei, P.R. China; Institute of Biochemistry and Biotechnology, TAIWAN

## Abstract

The effects of vitamin K2 on apoptosis in a variety of cancer cells have been well established in previous studies. However, the apoptotic effect of vitamin K2 on bladder cancer cells has not been evaluated. The aim of this study is to examine the apoptotic activity of Vitamin K2 in bladder cancer cells and investigate the underlying mechanism. In this study, Vitamin K2 induced apoptosis in bladder cancer cells through mitochondria pathway including loss of mitochondria membrane potential, cytochrome C release and caspase-3 cascade. Furthermore, the phosphorylation of c-Jun N-terminal kinase (JNK) and p38 MAPK was detected in Vitamin K2-treated cells and both SP600125 (an inhibitor of JNK) and SB203580 (an inhibitor of p38 MAPK) completely abolished the Vitamin K2-induced apoptosis and loss of mitochondria membrane potential. Moreover, the generation of reactive oxygen species (ROS) was detected in bladder cancer cells, upon treatment of vitamin K2 and the anti-oxidant N-acetyl cysteine (NAC) almost blocked the Vitamin K2-triggered apoptosis, loss of mitochondria membrane potential and activation of JNK and p38 MAPK. Taken together, these findings revealed that Vitamin K2 induces apoptosis in bladder cancer cells via ROS-mediated JNK/p38 MAPK and Mitochondrial pathways.

## Introduction

Bladder cancer is one of the most common carcinoma and ranks the ninth in worldwide cancer incidence. More than 12 million new cases arise each year globally. In particular, bladder cancer accounts for approximately 180,000 new cancer diagnosis and more than 50,000 deaths annually in the United States and European countries[1,2]. To cure human bladder cancer, traditional and current methods, such as radical cystectomy, chemotherapy, radiotherapy, concurrent chemotherapy and radotherapy, combination of radical cystectomy and chemotherapy and immunotherapy, are widely used[1,3–5]. However, these therapies usually encounter a variety of adverse effect such as distant metastasis, local recurrence, toxicity to health, low survival of patients and cost-effectiveness. Base on the above side effect and poor life quality of patients[4,6,7], new drugs are urgently required to treat bladder carcinoma.

Vitamin K is one of the fat-soluble vitamins which are indispensible to human health and rich in a variety of food. Usually, vitamin K exists in three forms including phylloquinone (VK1), menaquinone (VK2) and menadione (VK3). Predominant research on vitamin K has devoted to its role as a critical factor in blood coagulation, a cofactor in bone metabolism and prevention of cardiovascular calcification[8–10]. Recent years, a growing number of studies have revealed that vitamin K exhibited remarkable anti-proliferative effects on cancer cells.

Vitamin K2 (Menaquinone) is a series of vitamin K with multi-isoprene units at the 3-position of the naphthoquinone, which are named as MK-n by the number of the prenyl units[9,11]. For instance, MK-4, utilized in this study, is endowed with four isoprene units in its side chain. Original studies have discovered that vitamin K2 was produced by a vast array of bacteria and originally isolated from putrefied fishmeal as a product of microbial synthesis[9]. Recent studies have suggested vitamin K2 can actually be produced by animals and humans via conversion of other forms of vitamin K [12]. Furthermore, as the latest studies indicated, Menaquinone 4 (MK-4, one of vitamin K2 forms) was synthesized by UBIAD1, a geranylgeranyltransferase, in humans from the conversion of phylloquinone (VK1) and menadione (VK3) [12].

To date, abundant studies have shown that vitamin K2 can exhibit anticancer activity in various cancer cell lines, including leukemia, lung cancer, ovarian cancer, prostate cancer and heptocellular cancer [13–17]. Although some studies have revealed vitamin K2 exerted anticancer effect mainly by blocking the cell cycle at the G1 phase and inducing the caspase-3-mediated apoptosis, the detailed mechanism of anticancer effect of vitamin K2 remains unclear[17–19]. In this study, we demonstrated vitamin K2 induced apoptosis in human bladder cancer cells via generation of reactive oxygen species (ROS) which subsequently mediated MAPK and Mitochondrial pathways. Moreover, because vitamin K2 is ubiquitously produced in human and without adverse effects for clinical treatments, we adopted vitamin K2 treatment to nude mice bearing human bladder cancer cells and showed vitamin K2 sufficiently induced apoptosis of bladder cancer cells in vivo. This study was the first time to utilize vitamin K2 to treat human bladder cancer cells and demonstrated the detailed mechanism of anticancer activity of vitamin K2, which provide the basic theories for curing human bladder cancer.

## Materials and Methods

### Cell culture

The human bladder cancer cell lines (T24, J82 and EJ) and human normal cell lines (L02 and HEK293) were obtained from the American Type Culture Collection (Manassas, VA, USA). The T24, J82 and EJ cells were cultured in Minimum Essential Medium Eagle (MEM) supplemented with 10% Fetal Bovine Serum (FBS). While, the L02 and HEK293 cells were culture in Dulbecco’s modified Eagle’s medium (DMEM) supplemented with 10% Fetal Bovine Serum (FBS). All the cultures were maintained at 37°C in a humidified 5% CO_2_ incubator.

### Animal study

Twenty female BALB/c nude mice, 4- or 5-week old, were provided by experimental animal center (Tongji Medical college of Huazhong University of Science and Technology). Procedures and handing were strictly conducted in compliance with guidelines approved by the Science and Technology Department of Hubei province. All animal studies were approved by the Animal Experimentation Ethics Committee of Huazhong University of Science and Technology. All the efforts were made to minimize the animals’ suffering and to reduce the number of animals used.

### Drugs and reagents

Vitamin K2 was purchased from Sigma (USA), with carbon 82.6–84.9%, EmM 17.4–18.9 and completely soluted in ethonal. Vitamin K2 was dissolved in 99.9% ethanol at a stock concentration of 50 mM; it was then diluted to working concentration with MEM or DMEM. Ethanol was added to cultures at 0.1% (V/V) as a solvent control. 3-(4,5-dimethyl-2-thiazyl)-2,5-diphenyl-2H-tetrazolium bromide (MTT), N-acetyl cysteine (NAC) and Rhodamine 123 were purchased from Sigma Chemical Co. (St. Louis, MO). Annexin V-FITC (fluorescein isothiocyanate)/PI (propidium iodide) kit was purchased from BD Biosciences (San Jose, CA). The caspase-3 specific inhibitor Z-DEVD-FMK, JNK inhibitor SP600125 and p38 inhibitor SB203580 were purchase from Calbiochem (San Diego, CA). The antibodies against caspase-3, p-JNK, p38 and p-p38 were purchase from Cell signaling Technology. The antibodies against PARP, cytochrome C, COX IV, Bax, Puma, Bcl-2 and JNK were purchase from Proteintech. The antibodies against Actin was purchase from Santa Cruz.

### Cell viability assay

Cells were plated in 96-well plates at a density of approximately 1×10^5^ cells per well. Twenty four hours after plating, the cells were treated with vitamin K2. Cell viability was then evaluated using the MTT assay according to the manufacturer’s protocol. The number of viable cells was evaluated by uptake of MTT, assayed at 590nm. Assays were performed in triplicate on three independent experiments.

### Cell apoptosis assay

To analyze cellular apoptosis, cells were harvested, washed with PBS and resuspended in 500 μl of 1× binding buffer. The resuspended cells were then stained with Annexin V-FITC/PI and incubated in the dark for 15 minutes. The number of apoptotic cells was analyzed by flow cytometry (Beckman coulter FC500)

### TUNEL assay

DNA breaks were evaluated with an in situ cell death detecting kit (Roche Molecular Biochemicals, Basel, Switzerland), according to the manufacture’s instructions. Briefly, Cells were treated with the indicated concentration of vitamin K2 for 24 hours at 37°C in a 5% CO_2_ incubator, After incubation, cells were washed with PBS and fixed with 4% paraformaldehyde, then the cells were rinsed and subjected to TUNEL staining (terminal deoxynucleotidyl transferase dUTP nick end labeling). The apoptotic cells were observed under the fluorescence microscope (Olympus, Japan).

### Subcellular fraction

The protein in bladder cancer cells was separated into cytosolic and mitochondrial fraction using a special cytosolic and mitochondrial fraction kits (Beyotime, China), according to manufacture’s instructions. Briefly, Cells were harvested and mitochondrial isolation reagents were added and incubated on ice followed by centrifugation at 600g for 10 min. Supernatant was further centrifuged at 11,000g for 15 min. The mitochondrial fraction was contained in the pellets, while supernatant containing the cytosolic fraction.

### Western blot analysis

Cells were lysed with radioimmunoprecipitation (RIPA) buffer (Beyotime, Shanghai) supplemented with a protease inhibitor mixture tablet (Google Biology, Wuhan) for 30 minutes on ice. Total protein samples (40 μg) were then separated by SDS-PAGE (sodium lauryl sulfate (SDS)-polyacrylamide gel (PAGE)) and transferred to PVDF (polyvinylidenedifluoride) membranes (Millipore, USA). The membranes were subsequently blocked with 5% fat-free milk dissolved in Tris-Buffered Saline containing Tween-20 (TBST buffer) for 2 hours at room temperature and then probed with primary antibodies and incubated for overnight at 4°C. After incubation with horseradish peroxidase-conjugated secondary antibodies, The protein signals were detected using a chemiluminescence solution (ECL, Advansta, USA). Band intensity was quantified by Quantity one software (BioRad, USA).

### Cell mitochondria membrane potential assay

To evaluate the changes of mitochondria membrane potential, Rhodamine 123, a mitochondria specific dye, was used. Briefly, Cells were harvested and washed with PBS twice, then stained with 1.5 μM Rhodamine 123 and incubated at 37°C for 30 minutes. The cells were subsequently washed twice with cold PBS to remove the unbound dye. The mitochondria membrane potential was evaluated by the fluorescence of Rhodamine 123 under the flow cytometry with excitation and emission wavelengths of 488 and 525 nm (Beckman, FC500).

### Intracellular ROS detecting

To measure ROS generation, 2',7'-dichlorofluorescein-diacetate (DCFH-DA) was utilized. DCFH-DA, a cell membrane permeable dye, is converted to DCFH (a non-fluorescent cell membrane impermeable compound) by intracellular esterases and highly fluorescent DCF was produced by the oxidation of DCFH by intracellular ROS. Therefore, fluorescent DCF intensity is proportional to the amounts of intracellular ROS. Briefly, cells were harvested and stained with 10 μM DCFH-DA (Beyotime, China) for 30 minutes at 37°C, washed twice with PBS and then immediately analyzed by flow cytometry (Beckman, FC500). To observe intracellular ROS, cells were seeded on coverslips, treated with the indicated concentration of vitamin K2 for 24 hours, then stained with 10 μM DCFH-DA. Before DAPI staining, Cells were fixed in 4% paraformaldehyde, washed with PBS. Observing intracellular ROS was performed by confocal microscope (FV1000, Olympus).

### In vivo study

Human bladder cancer EJ cells (1×10^7^) suspended in PBS were injected subcutaneously into the lower right flank of each mouse. After 2 weeks, when tumors reached approximately 50 mm in diameter, the mice were randomly divided into two groups. 10 mice were used in each group. Treatment was 30mg/kg of vitamin K2 by directly injection at tumor each day as the experiment group, while treatment was the equivalent volume of PBS by directly injection at tumor per day as the control group. Tumor size was measured using a sliding caliper two times per week and the volume (mm^3^) was calculated by the formula (W^2^× L) /2. After 21 days, mice were sacrificed and tumors were excised and sectioned for caspas-3, HE staining and TUNEL assays.

### Statistical analyses

All the experiments were performed at least three times. The data were analyzed using GraphPad Prism software. The results are displayed as the mean ± standard deviation, and the differences were measured using Student’s t-test. Statistical significance was set at p<0.05.

## Results

### Vitamin K2 reduces bladder cancer cell viability

**Fig 1:**
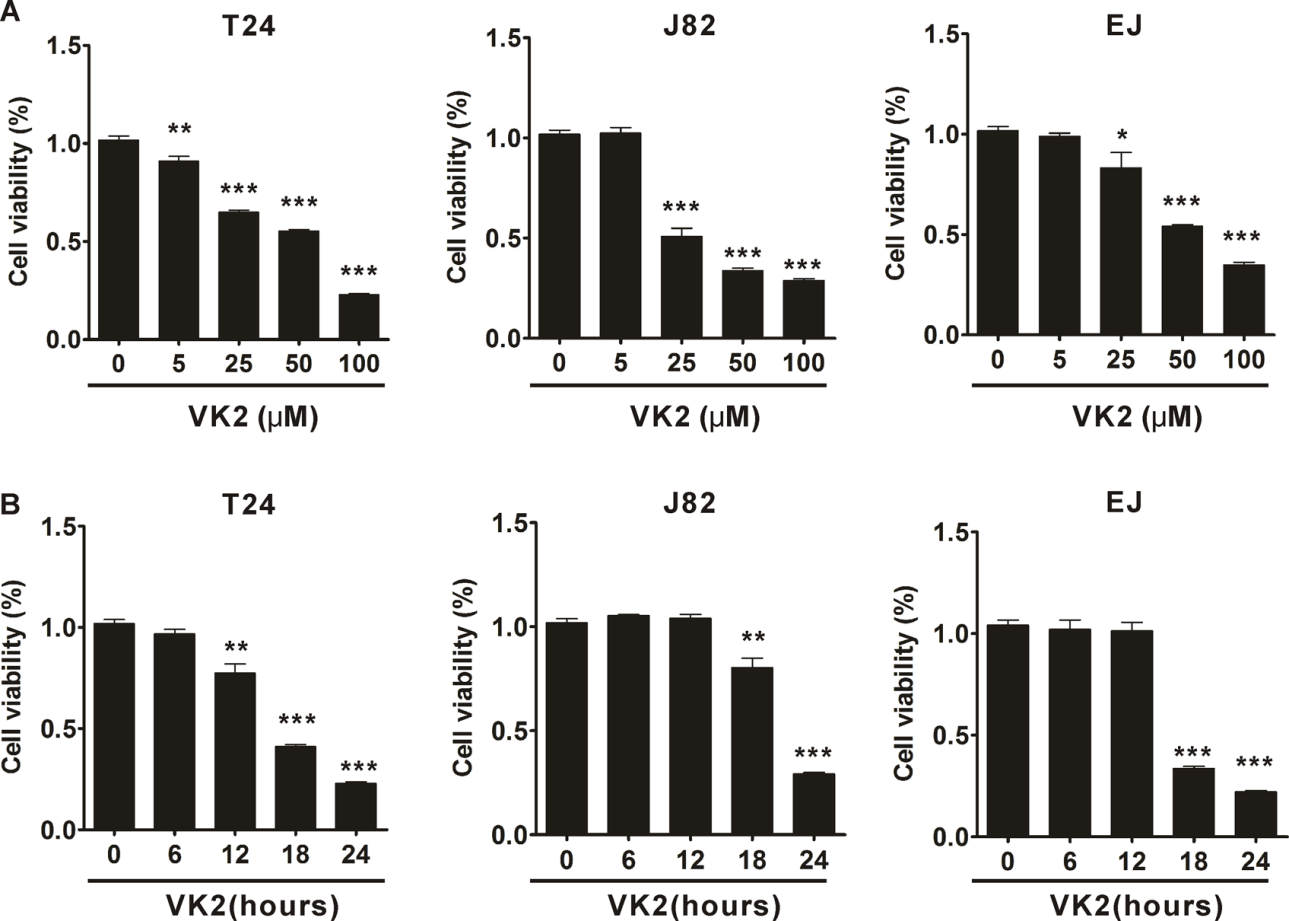
Effect of vitamin K2 on the viability of three human bladder cancer cells. (A). Vitamin K2 dose-dependently reduced the viability of human bladder cancer T24, J82 and EJ cells. Cells were treated different concentration of vitamin K2 for 24 hours, respectively and cell viability was measured by MTT assays. (B). Vitamin K2 time-dependently decreased the viability of T24, J82 and EJ cells. Cells were treated with 100 μM vitamin K2 for 0, 6, 12, 18 and 24 hours, respectively, and cell viability was evaluated by MTT assays. Data represent the mean ± SEM of three different experiments with triplicate sets in each assay. * P<0.05, ** P<0.01 and *** P<0.001 vs vitamin K2-untreated group.

To investigate the cell viability changes in human bladder cancer cells after treatment with vitamin K2, MTT assays were performed. Vitamin K2 significantly decreased the viability of human bladder cancer T24, J82 and EJ cells in a dose- and time-dependent manner. As shown in Fig 1A, T24, J82 and EJ cell viability was remarkably reduced following treatment with increasing concentrations of vitamin K2 (p<0.001). Similarly, viability of T24, J82 and EJ cells was significantly diminished with prolonged treatment with 100 μM vitamin K2 (p<0.001) (Fig 1B). On the other hand, viability of human normal cells (L02 and HEK293) was minimally affected after exposed to high concentration (100 μM) of Vitamin K2 (S1A Fig). These results suggest that vitamin K2 has anticancer activity in human bladder cancer cells, with low cytotoxic effect on human normal cells.

### Vitamin K2 induces significant apoptosis in human bladder cancer cells

**Fig 2:**
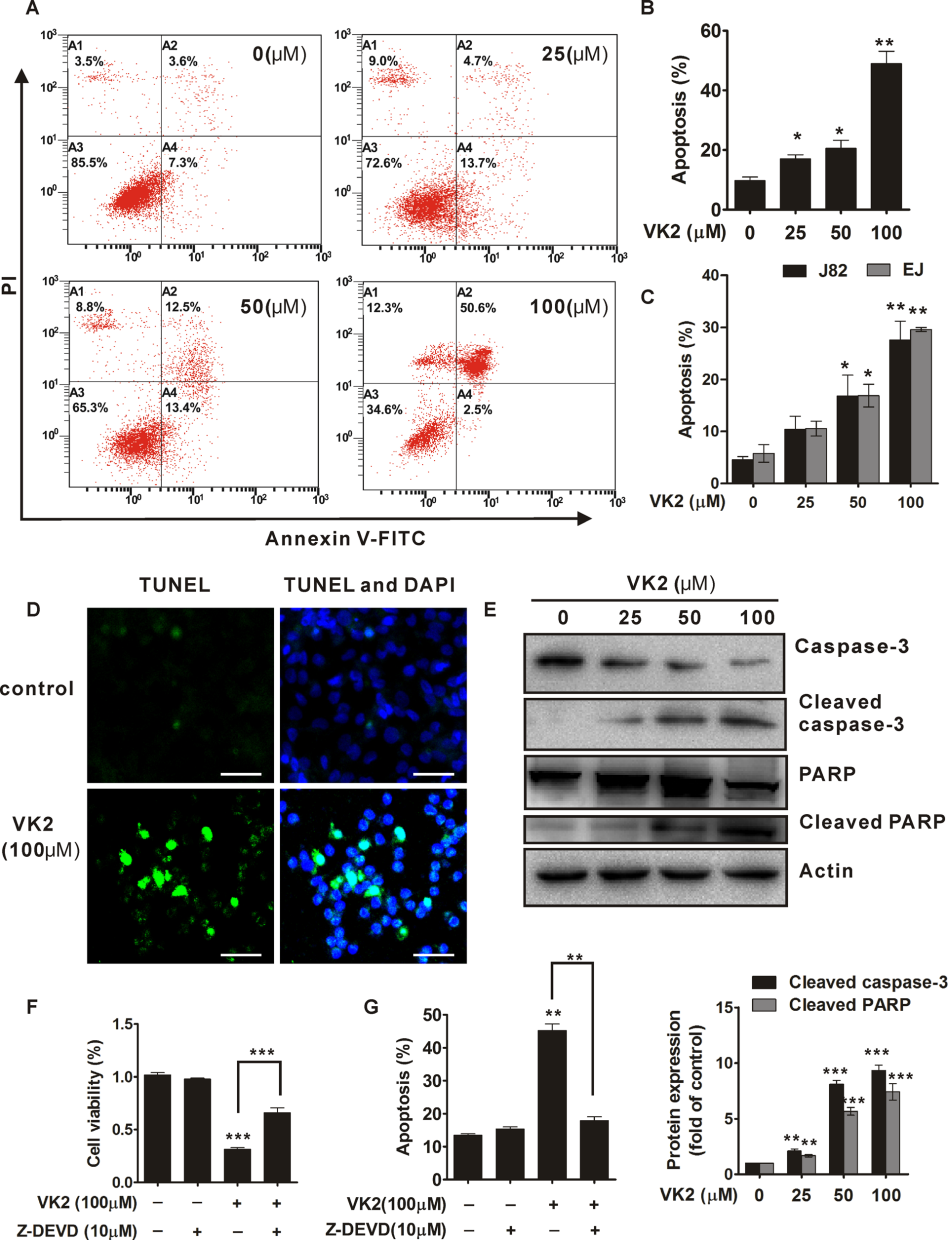
Vitamin K2 induced apoptotic cell death in human bladder cancer cells. (A). T24 cells were treated with the indicated concentration of vitamin K2 and the apoptosis was evaluated with Annexin V-FITC/PI dyes and measured by Flow cytometry. (B). The quantification of apoptotic death in vitamin K2-treated T24 cells. (C). Flow cytometry showed that vitamin K2 induced the apoptotic death in another two human bladder cancer J82 and EJ cells. (D). The effect of vitamin K2 on apoptosis in T24 cells was determined by TUNEL method using a detecting kit. Scale bar: 100μm (E). Western blots indicated that vitamin K2 induced activation of caspase-3 and cleavage of PARP in T24 cells. (F) Vitamin K2 inhibited the caspase-3-dependent viability of T24 cells by MTT assays. 10μM Z-DEVD-FMK, a caspase-3 inhibitor, was pretreated for 1 hours before exposure of 100 μM vitamin K2 to T24 cells for 24 hours. (G). Z-DEVD-FMK, a caspase-3 inhibitor, remarkably attenuated the apoptosis in vitamin K2-treated T24 cells. Cell apoptosis was evaluated with Annexin V-FITC/PI dyes and measured by Flow cytometry. * P<0.05, ** P<0.01 and *** P<0.001.

To evaluate the apoptotic effect of vitamin K2 on bladder cancer cells, T24, J82 and EJ cells were respectively exposed to indicated-concentration of vitamin K2 for 24 hours. As shown in Fig 2A, vitamin K2 remarkably triggered apoptosis in human bladder cancer T24 cells in a dose-dependent manner and approximately 50% of the cells occurred apoptosis after exposed to 100 μM vitamin K2 for 24 hours, compared with less than 10% in control group (0 μM vitamin K2) (Fig 2B). Similarly, J82 and EJ cells also underwent significant apoptosis after treatment with increasing concentration of vitamin K2, with approximately 30% of cells occurred apoptosis in 100 μM vitamin K2-treated group, compared with about 7.0% of cells in control group (Fig 2C and S2 Fig). In addition, TUNEL assays showed that DNA strands was dramatically broken in the T24 cells treated with 100μM vitamin K2 for 24 hours, compared with intact DNA strands in control group (Fig 2D). To further ascertain the apoptotic effect of vitamin K2 on human bladder cancer cells, caspase-3 and PARP, typical apoptotic markers, were measured by western blots. As shown in Fig 2E, cleaved caspase-3 and PARP were induced in vitamin K2 dose dependent-treated T24 cells, which indicated vitamin K2 indeed triggered apoptosis in human bladder cancer T24 cells. Next, to further confirm whether vitamin K2-induce T24 cell apoptosis was caspase-3 dependent, Z-DEVD-FMK, an inhibitor of caspase-3, was used. As indicated in MTT and apoptotic assay, the Z-DEVD-FMK significantly blocked the decreased viability of vitamin K2-treated T24 cells (Fig 2F) and abolished the vitamin K2-induced apoptosis in T24 cells (Fig 2G), which revealed that caspase-3 was involved in vitamin K2-induced apoptosis in T24 cells. To evaluate the apoptotic effect of vitamin K2 on human normal cells, HEK239T cells were utilized. As shown in S1B Fig, no significant apoptosis occurred in HEK239T cells after exposed to the indicated concentration of vitamin K2 for 24 hours. These results suggest that vitamin K2 undoubtedly triggers apoptosis in human bladder cancer cells, but not in human normal cells.

### Vitamin K2 induces mitochondria-related apoptosis in human bladder cancer cells

**Fig 3:**
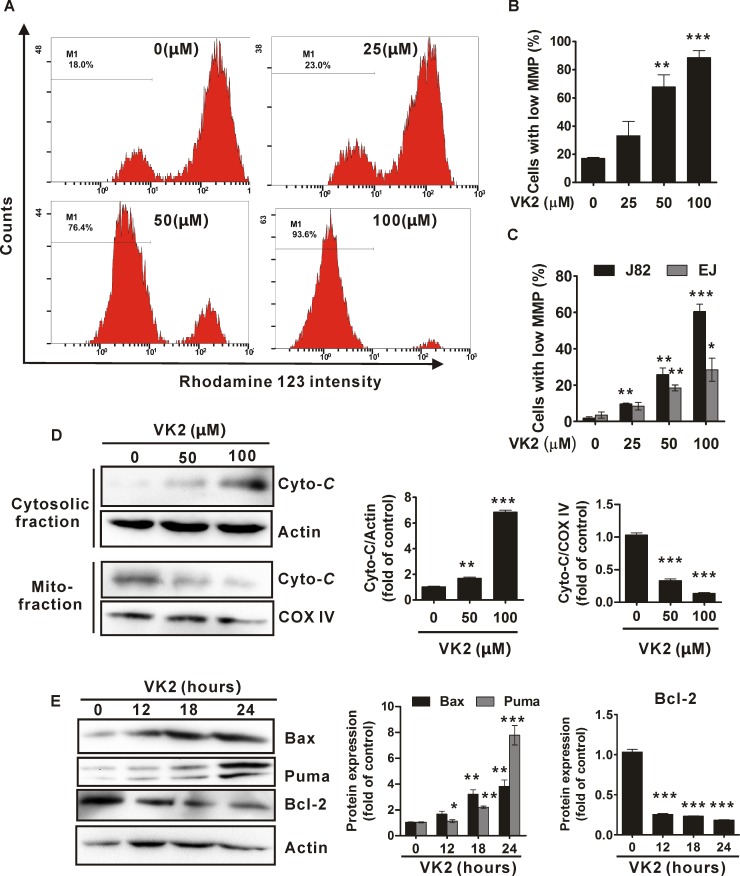
Vitamin K2 triggered mitochondria-related apoptosis in human bladder cancer cells. (A). T24 cells were treated with the indicated concentration of vitamin K2 for 24 hours and the disruption of mitochondria membrane potential was measured using a specific mitochondria dye Rhodamine 123 by flow cytometry. M1 stands for the percentage of cells with low mitochondria membrane potential. (B). Quantification of T24 cells with low mitochondria membrane potential. (C). J82 and EJ cells were treated the indicated concentration of vitamin K2 for 24 hours and cells with low mitochondria membrane potential was determined using the Rhodamine 123 dye by flow cytometry. (D). T24 cells were treated by the indicated concentration of vitamin K2 for 24 hours, then cells were harvested and separated into cytosolic and mitochondrial fractions using a commercial kit. The expression of cytochrome C in cytosol and mitochondria was evaluated by western blots. (E) T24 cells were treated with 100μM vitamin K2 for 0, 12, 18, 24 hours respectively, then the total proteins were isolated from the cells and the expression of Bax and Puma were analyzed by western blots. * P<0.05, ** P<0.01 and *** P<0.001.

To explore the underlying mechanism of vitamin K2-induced apoptosis in bladder cancer cells, we investigated whether mitochondria is associated with vitamin K2-induced apoptosis in human bladder cancer cells. As shown in Fig 3A, vitamin K2 remarkably disrupted the mitochondria membrane potential (MMP) of human bladder cancer T24 cells in a dose-dependent manner. As treatments with increasing concentration of vitamin K2 for 24 hours, a large number of T24 cells lost their MMP and approximately 90% of T24 cells had low MMP after exposed to vitamin K2 (100 μM) for 24 hours (Fig 3B). Similarly, vitamin K2 also caused significant collapse of MMP in J82 and EJ cells, another two human bladder cancer cells, in dose-dependent manners (Fig 3C). Moreover, the amount of cytochrome c in the mitochondrial fraction was reduced and conversely elevated in the cytosolic fraction after T24 cells were treated with vitamin K2 (50 μM and 100 μM) for 24 hours (Fig 3D). Next, we investigated whether Bcl-2 family proteins, such as Bax, Puma and Bcl-2, were implicated in the disruption of mitochondria membrane potential, upon vitamin K2 treatment. As shown in Fig 3E, vitamin K2 elevated the expression of Bax and Puma in T24 cells in a time-dependent manner. In contrast, the expression of Bcl-2, an anti-apoptotic protein, was diminished after prolonged treatment of vitamin K2. These results indicate that dysfunction of mitochondria is implicated in vitamin K2-induced apoptosis in human bladder cancer cells.

### Activation of JNK and p38 are required for vitamin K2-induced apoptosis in human bladder cancer cells

**Fig 4:**
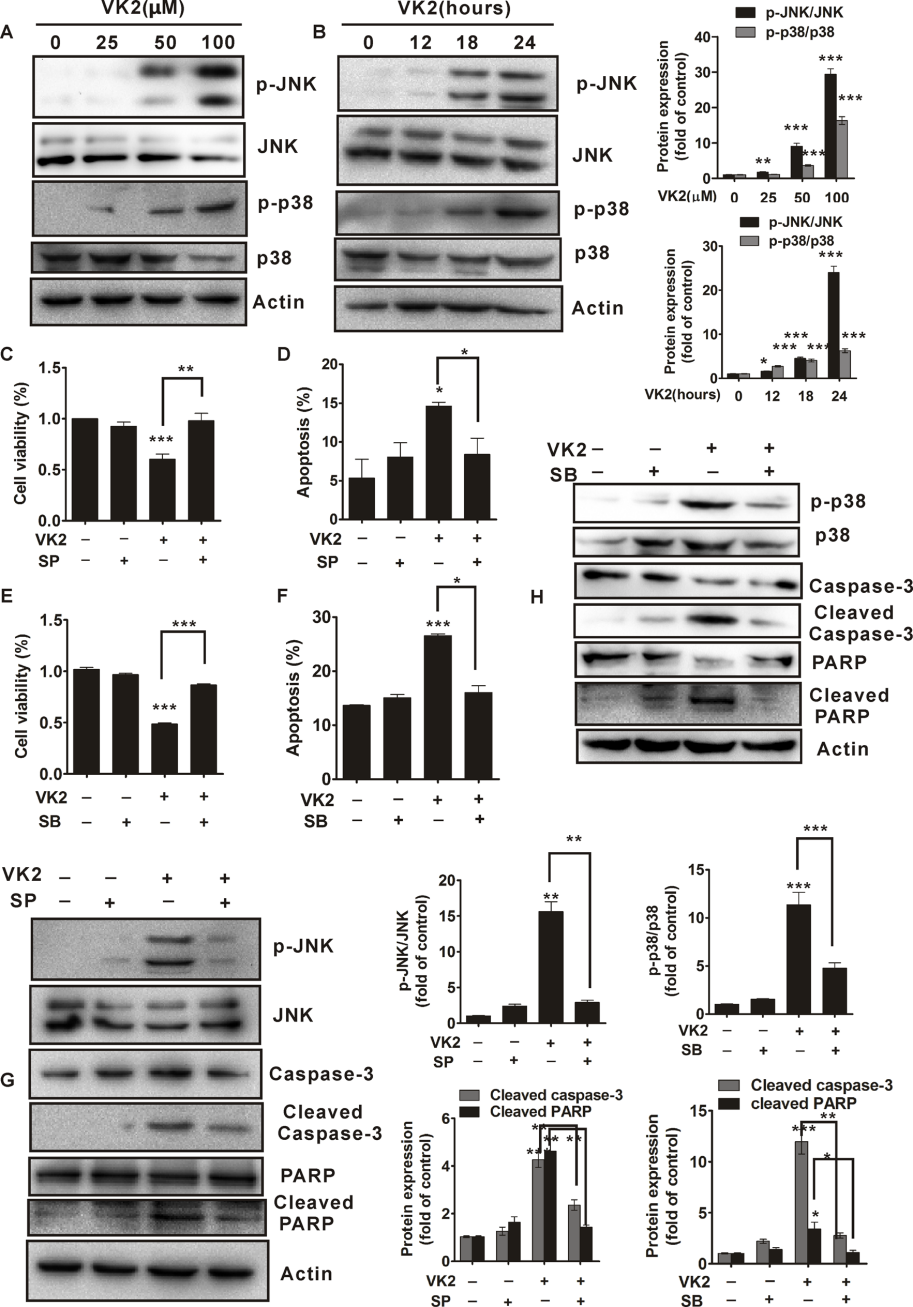
Activation of JNK/p38 is required for vitamin K2-triggered apoptosis in human bladder cancer T24 cells. (A). T24 cells were treated with the indicated concentration of vitamin K2 for 24 hours, then the total proteins were isolated from the cells and the phosphorylation of JNK/p38 was analyzed by western blots. (B). Western blots indicated that vitamin K2 at the concentration of 100 μM induced sustained phosphorylation of JNK/p38 in T24 cells. (C). T24 cells were treated with 40 μM SP600125(SP), a pharmacological inhibitor of JNK activation, for 1 hour before treatment with 100 μM vitamin K2 for 24 hours and cell viability was evaluated by MTT assays. (D). T24 cells were treated 40 μM SP600125(SP) for 1 hour before treatment with 100 μM vitamin K2 for 12 hours and apoptotic death was assessed by flow cytometry. (E). T24 cells were treated with 10 μM SB203580(SB), a pharmacological inhibitor of p38 activation, for 1 hour before treatment with 100 μM vitamin K2 for 24 hours, then cell viability was assessed by MTT assays. (F). T24 cells were pre-treated with 10 μM SB203580(SB) for 1 hour, then treated with 100 μM vitamin K2 for 12 hours and apoptotic death was determined by flow cytometry. (G). T24 cells were treated with 40μM SP600125(SP) for 1 hour prior to treatment with 100 μM vitamin K2 for 24 hours. The total proteins extracted from the cells were assessed by western blots. (H). T24 cells were treated with 10 μM SB203580(SB) for 1 hours before treatment with 100 μM vitamin K2 for 24 hours, the total protein was evaluated by western blots. * P<0.05, ** P<0.01 and *** P<0.001.

We next investigated whether MAPKs were involved in vitamin K2-induced apoptosis in bladder cancer cells. As shown in Fig 4A and 4B, vitamin K2 significantly induced phosphorylation of JNK and p38 in human bladder cancer T24 cells in a dose and time-dependent manner. To further confirm whether JNK and p38 activation contributed to vitamin K2-triggered apoptosis in human bladder cancer cells, SP600125 (a pharmacological inhibitor of JNK) and SB203580 (a pharmacological inhibitor of p38) were used. As shown in Fig 4C and 4D and S3A Fig, pretreatment of 40 μM SP600125 remarkably attenuated the decrease of cell viability and abrogated the apoptosis in T24 cells after exposed to 100 μM vitamin K2 for 24 hours. Moreover, phosphorylation of JNK, cleaved caspase-3 and PARP induced by vitamin K2 were significantly abolished by SP600125 (Fig 4G). These results indicate that JNK activation is involved in vitamin K2-induced apoptosis in human bladder cancer T24 cells. In addition, addition of 10 μM SB203580 significantly inhibited vitamin K2-induced the decrease of cell viability and blocked vitamin K2-triggered apoptosis in T24 cells (Fig 4E and 4F and S3B Fig). Furthermore, as shown in Fig 4H, SB203580 remarkably attenuated phosphorylation of p38 and inhibited cleaved caspase-3 and PARP in vitamin K2-treated T24 cells. Thus, active p38 is also associated with the apoptosis in vitamin K2-treated T24 cells.

### ROS generation is required for vitamin K2-triggered apoptosis in human bladder cancer cells

**Fig 5:**
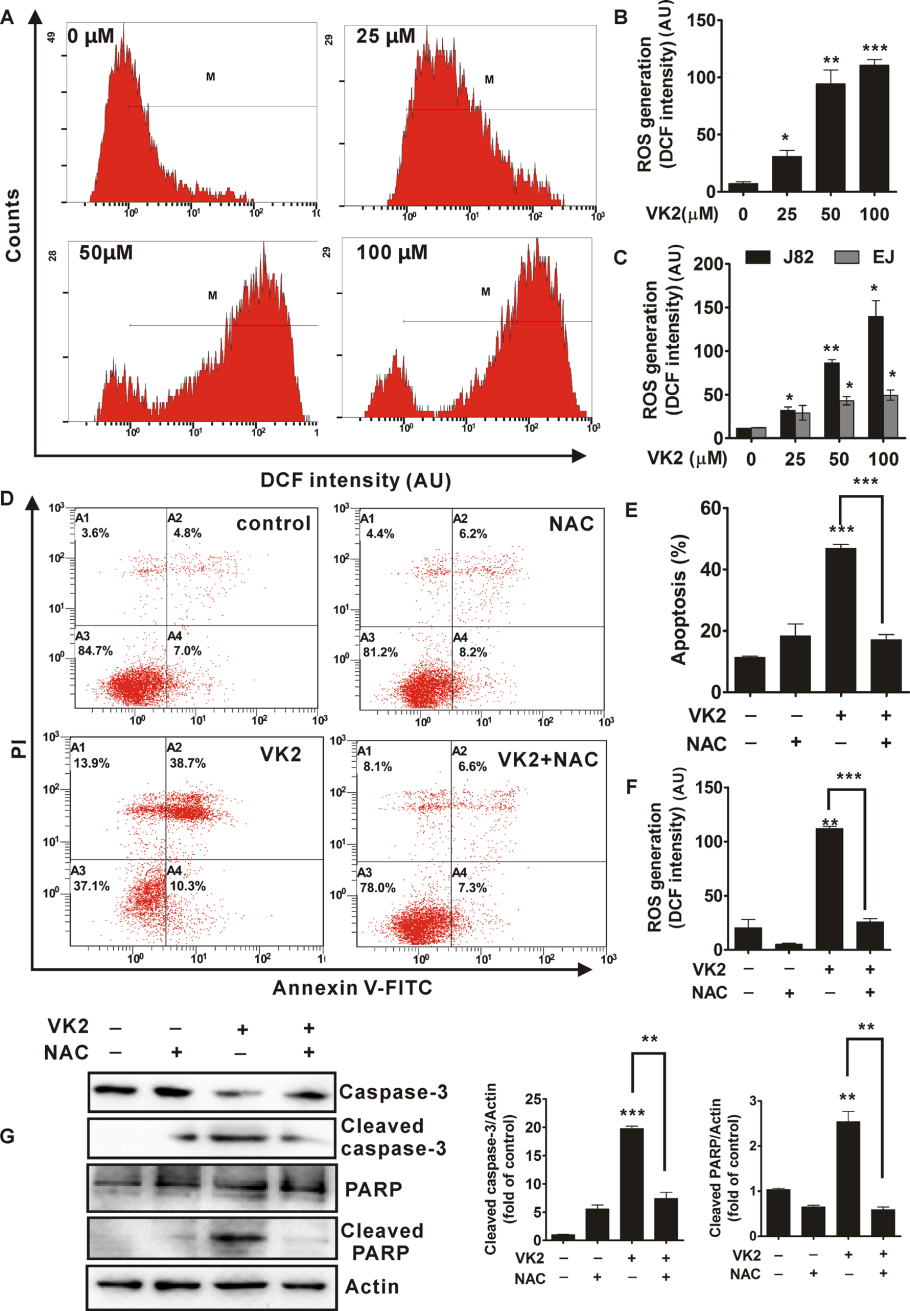
Vitamin K2 induced ROS-mediated apoptosis in human bladder cancer cells. (A). T24 cells were treated with the indicated concentration of vitamin K2 for 24 hours and the intracellular ROS generation was evaluated using the DCFH-DA probe by flow cytometry. M reflects the positive DCF fluorescence (B). Quantification of the intracellular ROS generation in vitamin K2-treated T24 cells. (C). J82 and EJ cells were treated with the indicated concentration of vitamin K2 for 24 hours and intracellular ROS generation was assessed by flow cytometry. (D and E). T24 cells were treated with 5mM antioxidant N-acetyl cysteine (NAC) for 1 hour before the treatment with or without 100 μM vitamin K2 for 24 hours and the apoptotic death was determined by flow cytometry. (F). T24 cells were treated with 5mM antioxidant NAC for 1 hour before the treatment with or without 100 μM vitamin K2 for 12 hours and intracellular ROS generation was evaluated using the DCFH-DA probe by flow cytometry. (G). Activation of caspase-3 and cleavage of PARP were analyzed by western blots after T24 cells were treated with 5mM NAC for 1 hour before the treatment with or without 100 μM vitamin K2 for 24 hours. * P<0.05, ** P<0.01 and *** P<0.001.

Given that Reactive Oxygen Species (ROS) are able to initiate various stimuli-induced apoptosis, Next, we assessed whether ROS was involved in vitamin K2-triggered apoptosis in human bladder cancer cells. As shown in Fig 5A and S4A Fig, after treatment with 100μM vitamin K2 for 24 hours, intracellular ROS was significantly generated in human bladder cancer T24 cells, compared with control group (0 μM vitamin K2). Moreover, vitamin K2 induced ROS over-production in T24 cells in a dose-dependent manner. As shown in Fig 5B, vitamin K2 at concentration of 50 μM and 100 μM, respectively, elevated ROS level almost by 7.0 and 10.0 fold of the vehicle-treated group in T24 cells. In addition, ROS levels were also remarkably enhanced in J82 and EJ cells, upon treatments with vitamin K2 in dose-dependent manner (Fig 5C). Since exposure of human bladder cancer cells to vitamin K2 triggered ROS generation, we next evaluated the role of ROS generation in vitamin K2-triggered apoptosis in human bladder cancer cells. As shown in Fig 5F and S4B Fig, N-acetyl cysteine (NAC), a ROS scavenger, completely blocked ROS generation in vitamin K2-treated T24 cells. In addition, NAC almost reversed the cell viability decrease (S4C Fig) and abolished the apoptosis in vitamin K2-treated T24 cells (Fig 5D and 5E). Furthermore, as shown in Fig 5G, cleavage of caspase-3 and PARP that induced by vitamin K2 were almost blocked by pre-treatment of NAC, suggesting NAC completely abolished the vitamin K2-triggered apoptosis. Collectively, ROS generation is required and plays an essential role in vitamin K2-triggered apoptosis in human bladder cancer cells.

### ROS mediates mitochondria dysfunction and regulates the activation of JNK and p38 in vitamin K2-treated human bladder cancer cells

**Fig 6:**
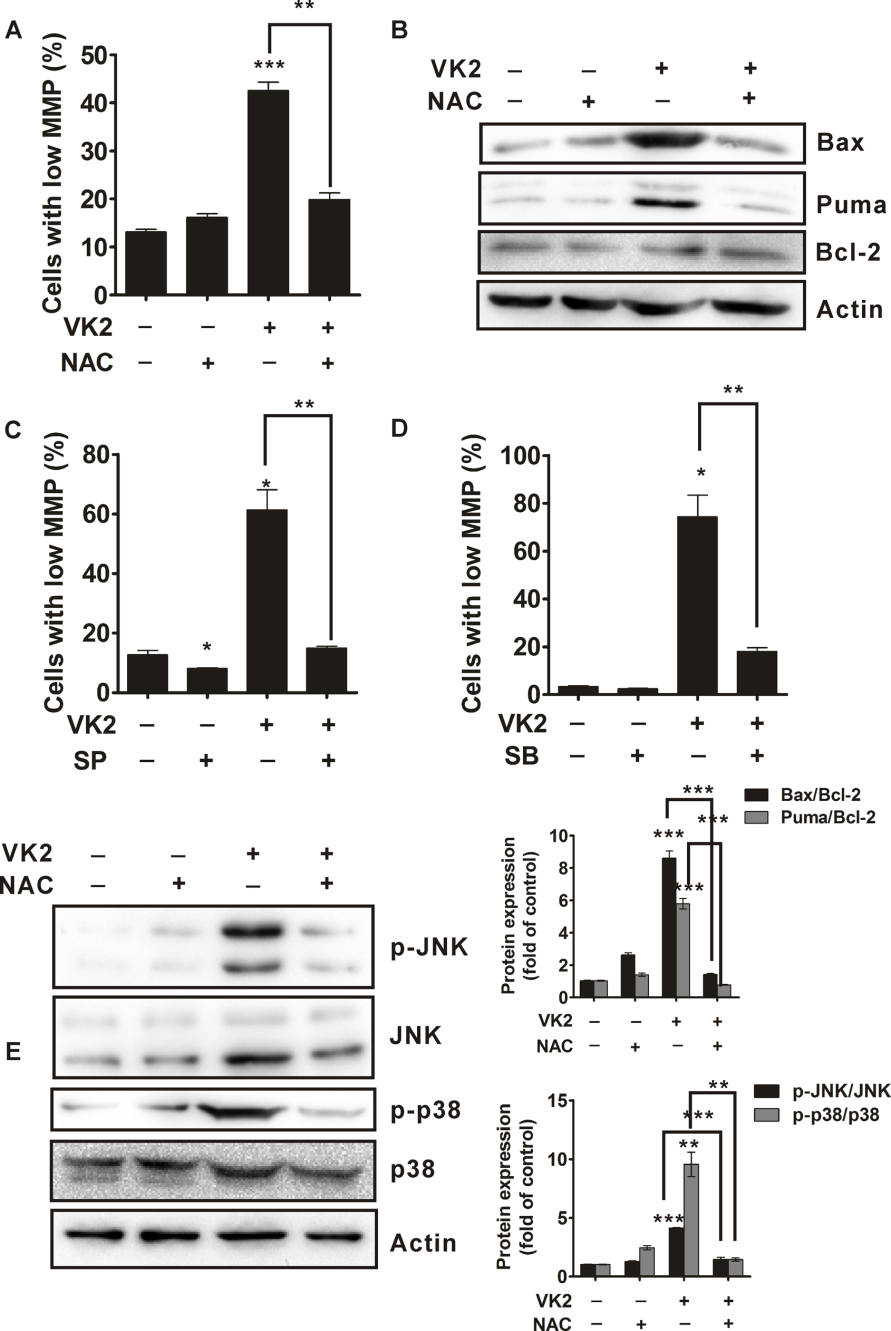
ROS mediated the mitochondria dysfunction and regulated activation of JNK/p38 in vitamin K2-triggered apoptosis of human bladder cancer T24 cells. (A). T24 cells were treated with 5mM antioxidant NAC for 1 hour prior to the treatment with or without 100 μM vitamin K2 for 24 hours, then the mitochondria membrane potential was assessed using the Rhodamine 123 dye by flow cytometry. (B). The expression of Bax, Puma and Bcl-2 were changed after treatment with 100 μM vitamin K2 for 24 hours in the present or absent of 5mM antioxidant N-acetyl cysteine (NAC) to human bladder cancer T24 cells. (C). T24 cells were treated 40 μM SP600125(SP) for 1 hour before treatment of 100 μM vitamin K2 for 24 hours, mitochondria membrane potential was evaluated using Rhodamine 123 dye by flow cytometry. (D). T24 cells were treated 10 μM SB203580(SB) for 1 hour before treatment of 100 μM vitamin K2 for 24 hours, mitochondria membrane potential was evaluated using Rhodamine 123 dye by flow cytometry. (E). T24 cells were treated with 5mM NAC for 1 hour before exposure to 100 μM vitamin K2 for 24 hours, then the total proteins were isolated from the cells and activation of JNK/p38 were determined by western blots. ** P<0.01 and *** P<0.001.

We next investigated the relationship between ROS generation and mitochondria dysfunction. As shown in Fig 6A, antioxidant NAC significantly attenuated the vitamin K2-induced disruption of mitochondria membrane potential in T24 cells, indicating that ROS generation was responsible for vitamin K2-induced mitochondria dysfunction. In addition, NAC remarkable inhibited vitamin K2-induced up-regulation of Bax and Puma (Fig 6B), suggesting that ROS regulated mitochondria dysfunction through ROS-mediated expression of Bax and Puma. Moreover, we continue to investigate whether activation of JNK and p38 were involved in Mitochondria dysfunction. Both SP600125 and SB203580 significantly inhibited the collapse of Mitochondria membrane potential, which reveals that activation of JNK and p38 contribute to Mitochondria dysfunction (Fig 6C and 6D). To verify the relationship between ROS and JNK/p38 in vitamin K2-induced apoptosis in bladder cancer cells, antioxidant NAC was used. As shown in Fig 6E, antioxidant NAC remarkably alleviated the vitamin K2-induced phosphorylation of JNK and p38, suggesting that ROS generation mediated activation of JNK and p38 in vitamin K2-treated T24 cells. These results indicate that vitamin K2 induces T24 cell apoptosis via ROS-JNK/p38-mediated mitochondria dysfunction.

### Vitamin K2 exerts the activity of inhibitory growth in xenografted nude mice model by causing apoptosis

**Fig 7:**
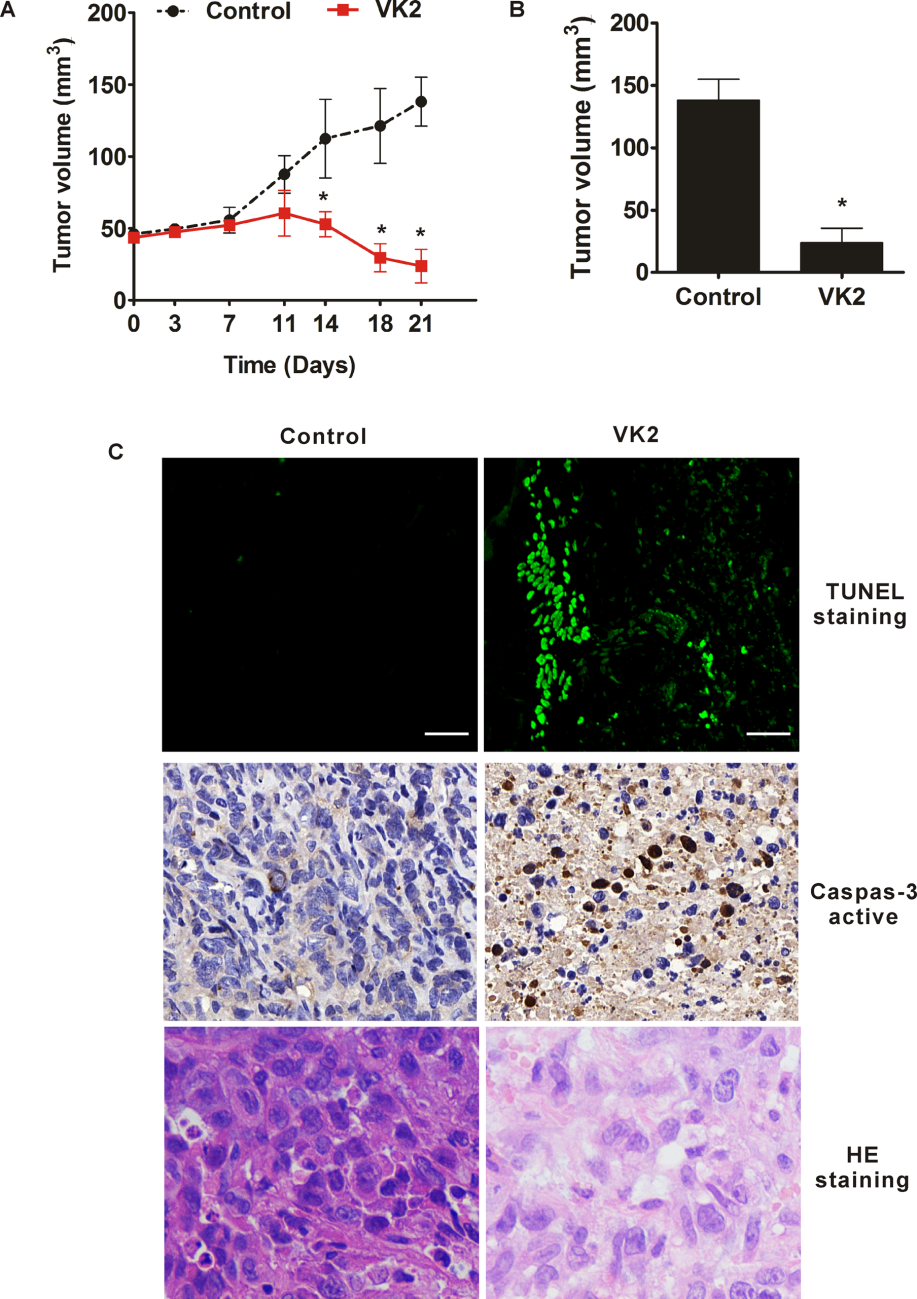
Vitamin K2 inhibited the tumor growth in mouse bearing human bladder cancer cells. **Nude mice with EJ transplant tumors were directly injected with 30 mg/kg vitamin K2 at tumors each day for 21 days**. (A). Tumor volume changed after administration with 30 mg/kg vitamin K2 everyday. (B). Measurement of tumor volume in mice after treatment with or without 30 mg/kg vitamin K2 each day for 21 days before sacrificed the nude mice. (C). After 21 days treatments, mice were sacrificed and tumors were excised to sections. Activation of caspase-3 in the sections was measured with the immuno-histo-chemistry method using antibody against caspase-3. Staining TUNEL and HE in the sections was measured by the commercial kits, respectively. Scale bar: 50μm.

To evaluate the effect of vitamin K2 on inhibitory growth in human bladder cancer cells in vivo, human bladder cancer EJ cells were injected subcutaneously into nude mice. When transplanted tumors reached a mean group size of approximately 50 mm^3^, mice were treated every day for 21 days by directly injection of 30 mg/kg vitamin K2 at tumors and directly injection of the equivalent volume of PBS as controls. As shown in Fig 7A and 7B, in nude mice, vitamin K2 remarkably inhibited the tumor growth and the tumor volume was gradually reduced after the 11^th^ day, compared with the sustained growth of control group. To determine whether the reduced tumor growth was due to the apoptotic effect of vitamin K2, we excised the tumors from the mice and sectioned for caspase-3 activity, TUNEL and HE staining assay. Compared with the control group, vitamin K2 induced activation of caspase-3 in tumor sections. Moreover, the TUNEL and HE staining assay showed the robust apoptosis in tumor sections from vitamin K2-treated mice, compared with the control group (Fig 7C). Taken together, vitamin K2 indeed inhibits the EJ cell growth in vivo by causing apoptotic death.

## Discussion

**Fig 8:**
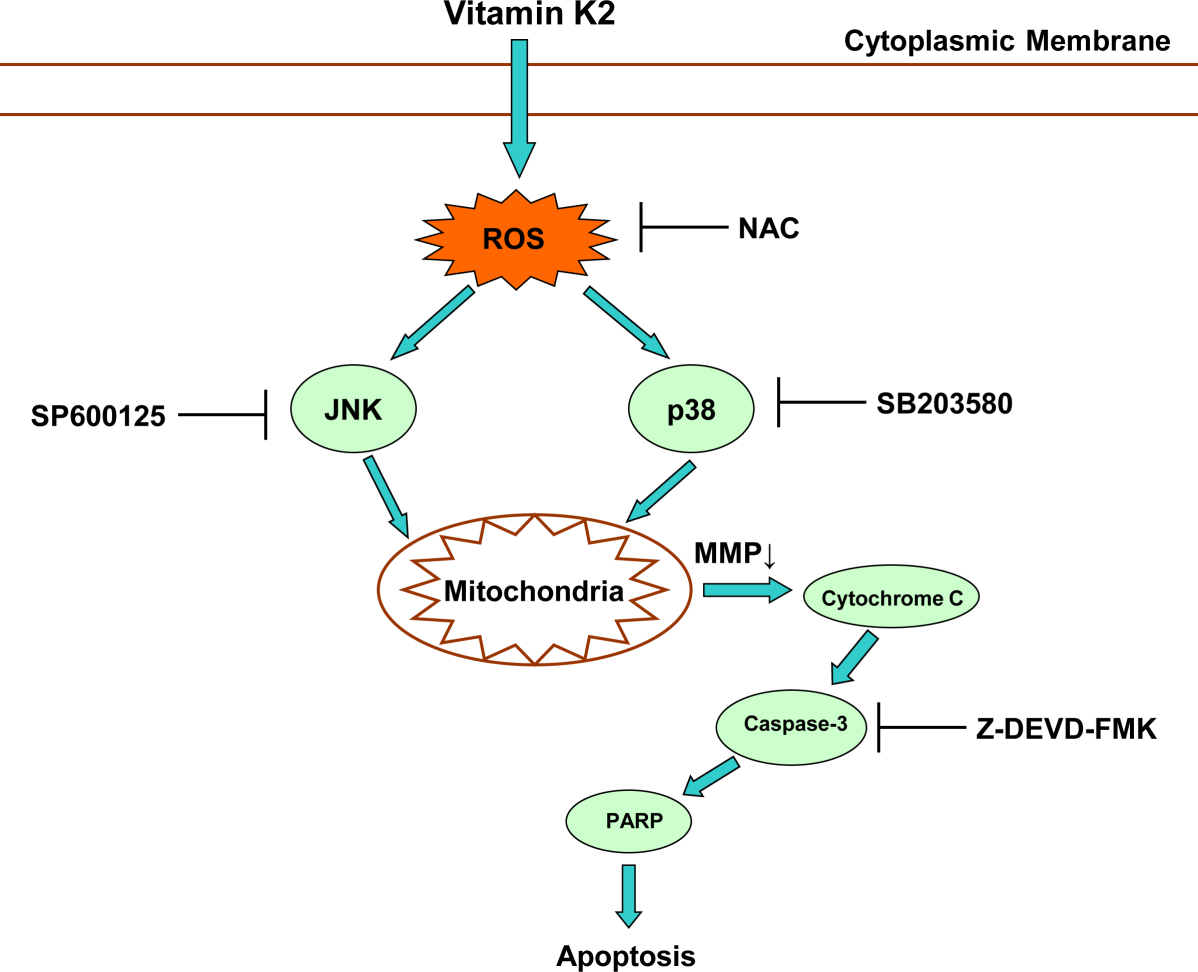
Schematic diagram of pathway involved in vitamin K2-induced apoptosis in human bladder cancer cells.

In this study, the anticancer effect of vitamin K2 on human bladder cancer cells was the first to demonstrate and the related mechanism was elucidated. As shown in Fig 8, vitamin K2 induces mitochondria-related apoptosis in human bladder cancer cells via ROS-JNK/p38 pathways, which explains the reason why vitamin K2 exerts anticancer activity in human bladder cancer cells.

As suggested in many recent studies, multiple clinical scenarios were employed to cure bladder cancer, including radical cystectomy, radiotherapy, chemotherapy, immunotherapy and so forth. In particular, radical cystectomy and chemotherapy are considered as effective therapeutic regimen to treat bladder cancer, however, they have many severe adverse effects, such as distant metastasis, local reccurence, toxicity to other normal organs and cost-effectiveness, which dramatically affect the life quality of patients[3,6,7]. Therefore, new therapeutics with less side effects to cure bladder cancer are greatly required. It is widely recognized that vitamin K2 is closely associated with the improvement of human health, including functions as cofactor for blood coagulation, bone metabolism and reduces the arterial calcium deposition avoiding vascular calcification[8,11]. Apart from these functions, interestingly, vitamin K2 also exerts potent anticancer activity in various cancer cells. Accumulating recent studies have documented that vitamin K2 induces growth suppression and apoptosis in a variety of cancer cells, including lung carcinomas, acute myeloid leukemia, ovarian cancer cells, prostate cancer cells and HCC cells[15,17–19]. Consistent with the previous studies, it was indicated in our results that vitamin K2 exerts anticancer activity in bladder cancer cells, including inhibits cell growth by reducing the cell viability and triggers cell apoptosis by DNA breaks, activation of caspase-3 and cleavage of PARP. Moreover, Z-DEVD-FMK, a pharmacological caspase-3 inhibitor, significantly reverses the vitamin K2-induced apoptosis in T24 cells, suggesting caspase-3 mediates vitamin K2-induced apoptosis in bladder cancer cells.

A growing number of studies have revealed that Mitochondria plays a pivotal role in regulating apoptotic signal pathways [20–22]. In this regard, targeting the mitochondria might be a novel strategy for cancer therapy. As indicated from the previous studies, the mechanism of mitochondria-mediated apoptosis mainly depends on the dysfunction of mitochondria including loss of mitochondria membrane potential and apoptotic factors, such as cytochrome C, AIF, and smac, release into cytosol, which subsequently, activates caspase cascade. Interestingly, in this study, the remarkable collapse of mitochondria membrane potential was displayed in vitamin K2-treated T24 cells and cytochrome C, in turn, released from the mitochondria to cytosol. These results suggest that vitamin K2 induces mitochondria-related apoptosis in human bladder cancer T24 cells. Recent studies have suggested that Bcl-2 family proteins are greatly responsible for mitochondria dysfunction[23–25]. Up-regulation of Bax, Bak and Puma (proapoptotic proteins of Bcl-2 family) due to cellular stress can directly or indirectly cause collapse of mitochondria membrane potential. Satoki et al. had recently reported that vitamin K2 induces up-regulation of Bax and Bak, which lead to loss of mitochondria membrane potential in Hela cells[25]. In accordance with the former studies, our results showed that up-regulation of Bax and puma was also induced in vitamin K2-treated T24 cells, which may be one of reasons that vitamin K2 caused loss of mitochondria membrane potential in T24 cells.

MAPKs are one of the sensors in response to extra-cellular stimuli and mediate the cellular signals[26–28]. ERK is usually associated with cell proliferation and growth. In contrast, JNK and p38 are induced by cellular stress and closely associated with cell death [29–31]. To elucidate the exact mechanism involving in vitamin K2-induced apoptosis in human bladder cancer cells, the effect of vitamin K2 on activation of MAPKs was examined. Our results showed vitamin K2 induced activation of JNK and p38 in human bladder cancer T24 cells. In addition, either the SP600125 (a JNK inhibitor), or SB203580 (a p38 inhibitor) completely blocked the vitamin K2-induced apoptosis in human bladder cancer T24 cells, suggesting activation of JNK and p38 are required and involved in vitamin K2-induced apoptosis in T24 cells. Furthermore, it is interesting that SP600125 as well as SB203580 remarkably alleviated the disruption of mitochondria membrane potential, which indicates JNK, as well as p38, contributes to vitamin K2-induced mitochondria dysfunction in human bladder cancer T24 cells.

There are increasing evidences indicating that reactive oxygen species (ROS) mediates the intracellular signal cascades and excessive ROS production leads to intracellular stress, mitochondria dysfunction and ultimately cell apoptosis or necrosis [32–35]. In this study, vitamin K2 induced ROS generation in human bladder cancer cells in a dose-dependent manner. Moreover, antioxidant NAC significantly abolished the apoptosis and collapse of mitochondria membrane potential in vitamin K2-treated T24 cells. These results suggest that ROS remarkably mediates the mitochondria-related apoptosis in vitamin K2-treated T24 cells. Recent studies have elucidated that ROS mediated MAPKs activation in various stimuli-triggered cell apoptosis[26,31,36]. Concordantly, in our hands, antioxidant NAC significantly inhibited phosphorylation of JNK and p38, suggesting that ROS generation activates the JNK and p38, which is supposed to the upstream of vitamin K2-induced apoptotic pathway in human bladder cancer T24 cells. Growing evidence has indicated that many chemotherapeutic agents exhibit anticancer activity in numerious cancer cells by inducing ROS generation, suggesting that vitamin K2, in some extent like chemotherapeutic drugs, exerts anticancer activity in bladder cancer cells by providing oxidative stress.

In vivo study, we investigated the effect of vitamin K2 on the growth of human bladder cancer cells. As the results shown, vitamin K2 indeed inhibits the tumor growth in xenografte nude mice. In addition, we further determined that the inhibition of tumor growth was mainly due to vitamin K2-induced apoptotic cell death. These results indicate vitamin K2 is able to induce apoptosis in human bladder cancer cells in vivo. Furthermore, it is indicated from the latest studies that vitamin K2 is not applied in clinical therapy for cancer because of its insufficient strong activity to cancer. Considering the current clinical concerns, the method of directly injection of vitamin K2 at tumor was employed in our studies. Interestingly, treatment by directly injection of vitamin K2 into the tumors is a highly efficient method to kill the human bladder cancer cells in vivo, which maybe provide a new sight for clinical research.

In conclusion, our results demonstrated that vitamin K2 was able to induce mitochondria-related apoptosis in human bladder cancer cells via ROS-JNK/p38 MAPK signal pathways, which indicated a detailed mechanism of the anticancer activity of vitamin K2 in human bladder cancer cells. In addition, vitamin K2 also suppresses the growth of human bladder cancer cells in nude mice, which was further confirmed by vitamin K2-induced apoptosis. Considering the potent apoptotic effect on human bladder cancer cells but not on human normal cells, vitamin K2 will become a promising anticancer agent to cure human bladder cancer in future.

## Supporting Information

S1 Fig Vitamin K2 had no siginificant effect on cell viability or apoptosis in human normal cells. (TIF)

S2 Fig Vitamin K2 induced apoptosis in human bladder cancer J82 and EJ cells (TIF)

S3 Fig Vitamin K2 induced JNK/p38-mediated apoptosis in human bladder cancer T24 cells. (TIF)

S4 Fig Intracellular ROS is generated and responsible for the viability decrease in vitamin K2-treated T24 cells. (TIF)

## References

[1] Witjes JA. Bladder cancer in 2015: Improving indication, technique and outcome of radical cystectomy. Nat Rev Urol. 2016; 13: 74–76. 10.1038/nrurol.2015.272 26597614

[2] Hermans TJ, Mertens LS, van Rhijn BW. Re: Trends in the Use of Perioperative Chemotherapy for Localized and Locally Advanced Muscle-invasive Bladder Cancer: A Sign of Changing Tides. Eur Urol. 2016; 69: 1156–1157. 10.1016/j.eururo.2016.02.023 27302138

[3] Stevenson SM, Danzig MR, Ghandour RA, Deibert CM, Decastro GJ, Benson MC, et al Cost-effectiveness of neoadjuvant chemotherapy before radical cystectomy for muscle-invasive bladder cancer. Urol Oncol. 2014; 32: 1172–1177. 10.1016/j.urolonc.2014.05.001 24998787

[4] Feuerstein MA, Goenka A. Quality of Life Outcomes for Bladder Cancer Patients Undergoing Bladder Preservation with Radiotherapy. Curr Urol Rep. 2015; 16:75 10.1007/s11934-015-0547-1 26343030

[5] Schepisi G, Santoni M, Massari F, Gurioli G, Salvi S, Conteduca V, et al Urothelial Cancer: Inflammatory Mediators and Implications for Immunotherapy. BioDrugs. 2016: PMID: 27177757 10.1007/s40259-016-0176-3 27177757

[6] Juffs HG, Moore MJ, Tannock IF. The role of systemic chemotherapy in the management of muscle-invasive bladder cancer. Lancet Oncol. 2002; 3: 738–747. 10.1016/S1470-2045(02)00930-0 12473515

[7] Hafeez S, Huddart R. Selective organ preservation for the treatment of muscle-invasive transitional cell carcinoma of the bladder: a review of current and future perspectives. Expert Rev Anticancer Ther. 2014; 14: 1429–1443. 10.1586/14737140.2014.953938 25263197

[8] O'Keefe JH, Bergman N, Carrera-Bastos P, Fontes-Villalba M, DiNicolantonio JJ, Cordain L. Nutritional strategies for skeletal and cardiovascular health: hard bones, soft arteries, rather than vice versa. Open Heart. 2016; 3: e000325 10.1136/openhrt-2015-000325 27042317 PMC4809188

[9] Lamson DW, Plaza SM. The anticancer effects of vitamin K. Altern Med Rev. 2003; 8: 303–318 12946240

[10] Ebina K, Noguchi T, Hirao M, Kaneshiro S, Tsukamoto Y, Yoshikawa H. Comparison of the effects of 12 months of monthly minodronate monotherapy and monthly minodronate combination therapy with vitamin K2 or eldecalcitol in patients with primary osteoporosis. J Bone Miner Metab. 2016; 34: 243–250. 10.1007/s00774-015-0710-2 26303222

[11] Shearer MJ, Newman P. Recent trends in the metabolism and cell biology of vitamin K with special reference to vitamin K cycling and MK-4 biosynthesis. J Lipid Res. 2014; 55: 345–362. 10.1194/jlr.R045559 24489112 PMC3934721

[12] Nakagawa K, Hirota Y, Sawada N, Yuge N, Watanabe M, Uchino Y, et al Identification of UBIAD1 as a novel human menaquinone-4 biosynthetic enzyme. Nature. 2010; 468: 117–121. 10.1038/nature09464 20953171

[13] Yoshida T, Miyazawa K, Kasuga I, Yokoyama T, Minemura K, Ustumi K, et al Apoptosis induction of vitamin K2 in lung carcinoma cell lines: the possibility of vitamin K2 therapy for lung cancer. Int J Oncol. 2003; 23: 627–632. 10.3892/ijo.23.3.627 12888897

[14] Yaguchi M, Miyazawa K, Katagiri T, Nishimaki J, Kizaki M, Tohyama K, et al Vitamin K2 and its derivatives induce apoptosis in leukemia cells and enhance the effect of all-trans retinoic acid. Leukemia. 1997; 11: 779–787. 9177427 10.1038/sj.leu.2400667

[15] Wei G, Wang M, Hyslop T, Wang Z, Carr BI. Vitamin K enhancement of sorafenib-mediated HCC cell growth inhibition in vitro and in vivo. Int J Cancer. 2010; 127: 2949–2958. 10.1002/ijc.25498 21351273 PMC2955185

[16] Miyazawa K, Yaguchi M, Funato K, Gotoh A, Kawanishi Y, Nishizawa Y, et al Apoptosis/differentiation-inducing effects of vitamin K2 on HL-60 cells: dichotomous nature of vitamin K2 in leukemia cells. Leukemia. 2001; 15: 1111–1117. 11455981 10.1038/sj.leu.2402155

[17] Samykutty A, Shetty AV, Dakshinamoorthy G, Kalyanasundaram R, Zheng G, Chen A, et al Vitamin k2, a naturally occurring menaquinone, exerts therapeutic effects on both hormone-dependent and hormone-independent prostate cancer cells. Evid Based Complement Alternat Med. 2013; 2013: 287358 10.1155/2013/287358 24062781 PMC3767046

[18] Tokita H, Tsuchida A, Miyazawa K, Ohyashiki K, Katayanagi S, Sudo H, et al Vitamin K2-induced antitumor effects via cell-cycle arrest and apoptosis in gastric cancer cell lines. Int J Mol Med. 2006; 17: 235–243. 10.3892/ijmm.17.2.235 16391821

[19] Matsumoto K, Okano J, Nagahara T, Murawaki Y. Apoptosis of liver cancer cells by vitamin K2 and enhancement by MEK inhibition. Int J Oncol. 2006; 29: 1501–1508. 10.3892/ijo.29.6.1501 17088989

[20] Park GB, Kim YS, Lee HK, Song H, Kim S, Cho DH, et al Reactive oxygen species and p38 MAPK regulate Bax translocation and calcium redistribution in salubrinal-induced apoptosis of EBV-transformed B cells. Cancer Lett. 2011; 313: 235–248. 10.1016/j.canlet.2011.09.011 22056078

[21] Yang CR, Liao WS, Wu YH, Murugan K, Chen C, Chao JI. CR108, a novel vitamin K3 derivative induces apoptosis and breast tumor inhibition by reactive oxygen species and mitochondrial dysfunction. Toxicol Appl Pharmacol. 2013; 273: 611–622. 10.1016/j.taap.2013.10.007 24128853

[22] Shibayama-Imazu T, Sonoda I, Sakairi S, Aiuchi T, Ann WW, Nakajo S, et al Production of superoxide and dissipation of mitochondrial transmembrane potential by vitamin K2 trigger apoptosis in human ovarian cancer TYK-nu cells. Apoptosis. 2006; 11: 1535–1543. 10.1007/s10495-006-7979-5 16763728

[23] Yu J, Zhang L. PUMA, a potent killer with or without p53. Oncogene. 2008; 27 Suppl 1: S71–83. doi: 10.1038/onc.2009.45PMID: 860432 19641508 PMC2860432

[24] Zhang D, Armstrong JS. Bax and the mitochondrial permeability transition cooperate in the release of cytochrome c during endoplasmic reticulum-stress-induced apoptosis. Cell Death Differ. 2007; 14: 703–715. 10.1038/sj.cdd.4402072 17170750

[25] Karasawa S, Azuma M, Kasama T, Sakamoto S, Kabe Y, Imai T, et al Vitamin K2 covalently binds to Bak and induces Bak-mediated apoptosis. Mol Pharmacol. 2013; 83: 613–620. 10.1124/mol.112.082602 23229512

[26] Shi Y, Nikulenkov F, Zawacka-Pankau J, Li H, Gabdoulline R, Xu J, et al ROS-dependent activation of JNK converts p53 into an efficient inhibitor of oncogenes leading to robust apoptosis. Cell Death Differ. 2014; 21: 612–623. 10.1038/cdd.2013.186 24413150 PMC3950324

[27] Song IS, Jun SY, Na HJ, Kim HT, Jung SY, Ha GH, et al Inhibition of MKK7-JNK by the TOR signaling pathway regulator-like protein contributes to resistance of HCC cells to TRAIL-induced apoptosis. Gastroenterology. 2012; 143: 1341–1351. 10.1053/j.gastro.2012.07.103 22841785

[28] Wang H, Jiang D, Liu J, Ye S, Xiao S, Wang W, et al Compound K induces apoptosis of bladder cancer T24 cells via reactive oxygen species-mediated p38 MAPK pathway. Cancer Biother Radiopharm. 2013; 28: 607–614. 10.1089/cbr.2012.1468 23895116 PMC3777554

[29] Javadov S, Jang S, Agostini B. Crosstalk between mitogen-activated protein kinases and mitochondria in cardiac diseases: therapeutic perspectives. Pharmacol Ther. 2014; 144: 202–225. 10.1016/j.pharmthera.2014.05.013 24924700 PMC4185221

[30] Krilleke D, Ucur E, Pulte D, Schulze-Osthoff K, Debatin KM, Herr I. Inhibition of JNK signaling diminishes early but not late cellular stress-induced apoptosis. Int J Cancer. 2003; 107: 520–527. 10.1002/ijc.11331 14520687

[31] Xiong XX, Liu JM, Qiu XY, Pan F, Yu SB, Chen XQ. Piperlongumine induces apoptotic and autophagic death of the primary myeloid leukemia cells from patients via activation of ROS-p38/JNK pathways. Acta Pharmacol Sin. 2015; 36: 362–374. 10.1038/aps.2014.141 25619389 PMC4349924

[32] Santabarbara-Ruiz P, Lopez-Santillan M, Martinez-Rodriguez I, Binagui-Casas A, Perez L, Milan M, et al ROS-Induced JNK and p38 Signaling Is Required for Unpaired Cytokine Activation during Drosophila Regeneration. PLoS Genet. 2015; 11: e1005595 10.1371/journal.pgen.1005595 26496642 PMC4619769

[33] Ghavami S, Kerkhoff C, Los M, Hashemi M, Sorg C, Karami-Tehrani F. Mechanism of apoptosis induced by S100A8/A9 in colon cancer cell lines: the role of ROS and the effect of metal ions. J Leukoc Biol. 2004; 76: 169–175. 10.1189/jlb.0903435 15075348

[34] Vaseva AV, Marchenko ND, Ji K, Tsirka SE, Holzmann S, Moll UM. p53 opens the mitochondrial permeability transition pore to trigger necrosis. Cell. 2012; 149: 1536–1548. 10.1016/j.cell.2012.05.014 22726440 PMC3383624

[35] Latimer HR, Veal EA. Peroxiredoxins in Regulation of MAPK Signalling Pathways; Sensors and Barriers to Signal Transduction. Mol Cells. 2016; 39: 40–45. 10.14348/molcells.2016.2327 26813660 PMC4749872

[36] Li S, Dong P, Wang J, Zhang J, Gu J, Wu X, et al Icariin, a natural flavonol glycoside, induces apoptosis in human hepatoma SMMC-7721 cells via a ROS/JNK-dependent mitochondrial pathway. Cancer Lett. 2010; 298: 222–230. 10.1016/j.canlet.2010.07.009 20674153

