# Supplementary figures and images for: Vitamin K2 Induces Mitochondria-Related Apoptosis in Human Bladder Cancer Cells via ROS and JNK/p38 MAPK Signal Pathways
Source: PLoS One. 2016 Aug 29;11(8):e0161886. doi: 10.1371/journal.pone.0161886 (PMC5003392; doi:10.1371/journal.pone.0161886)

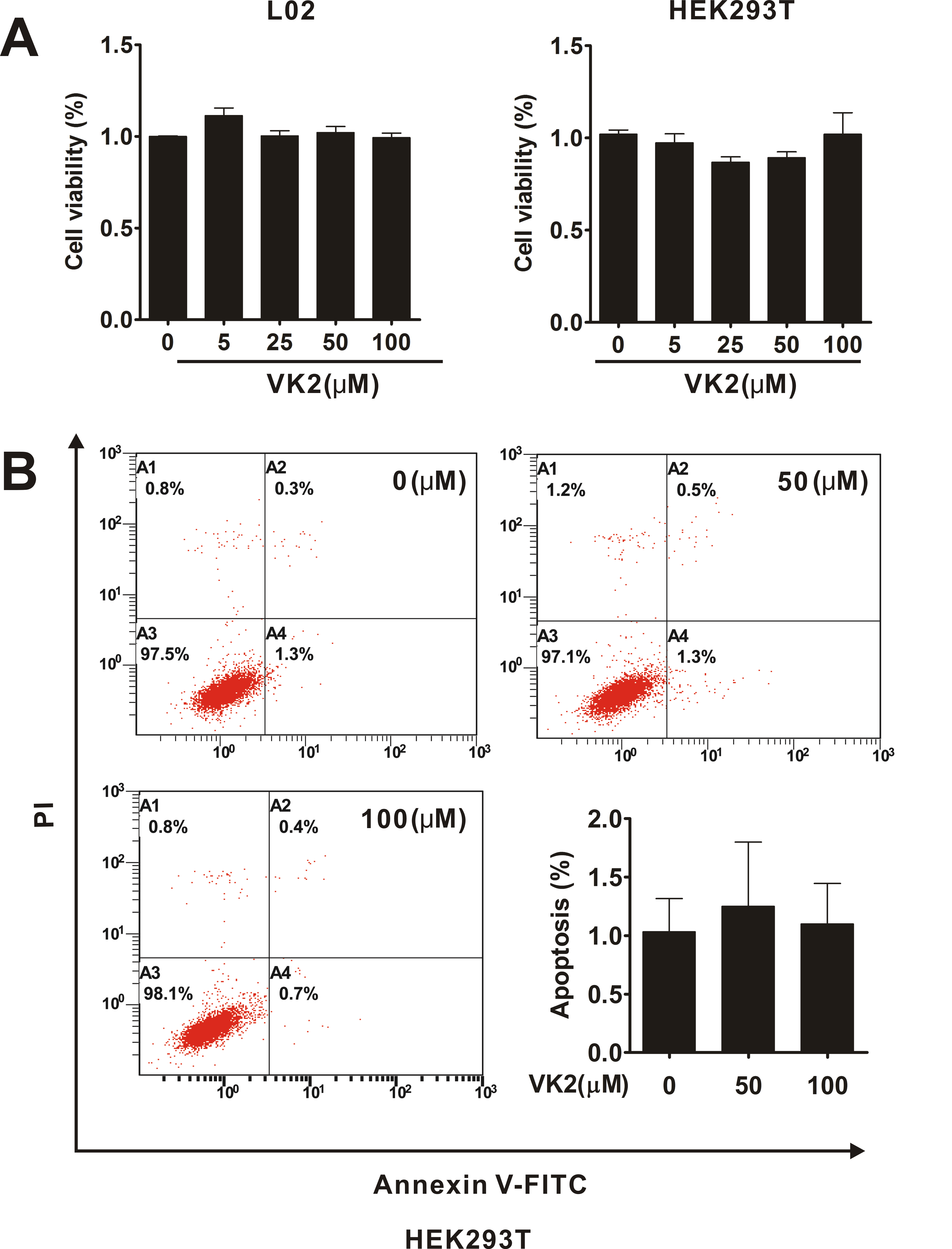

Supplement: S1 Fig — (TIF) [file pone.0161886.s001.tif]

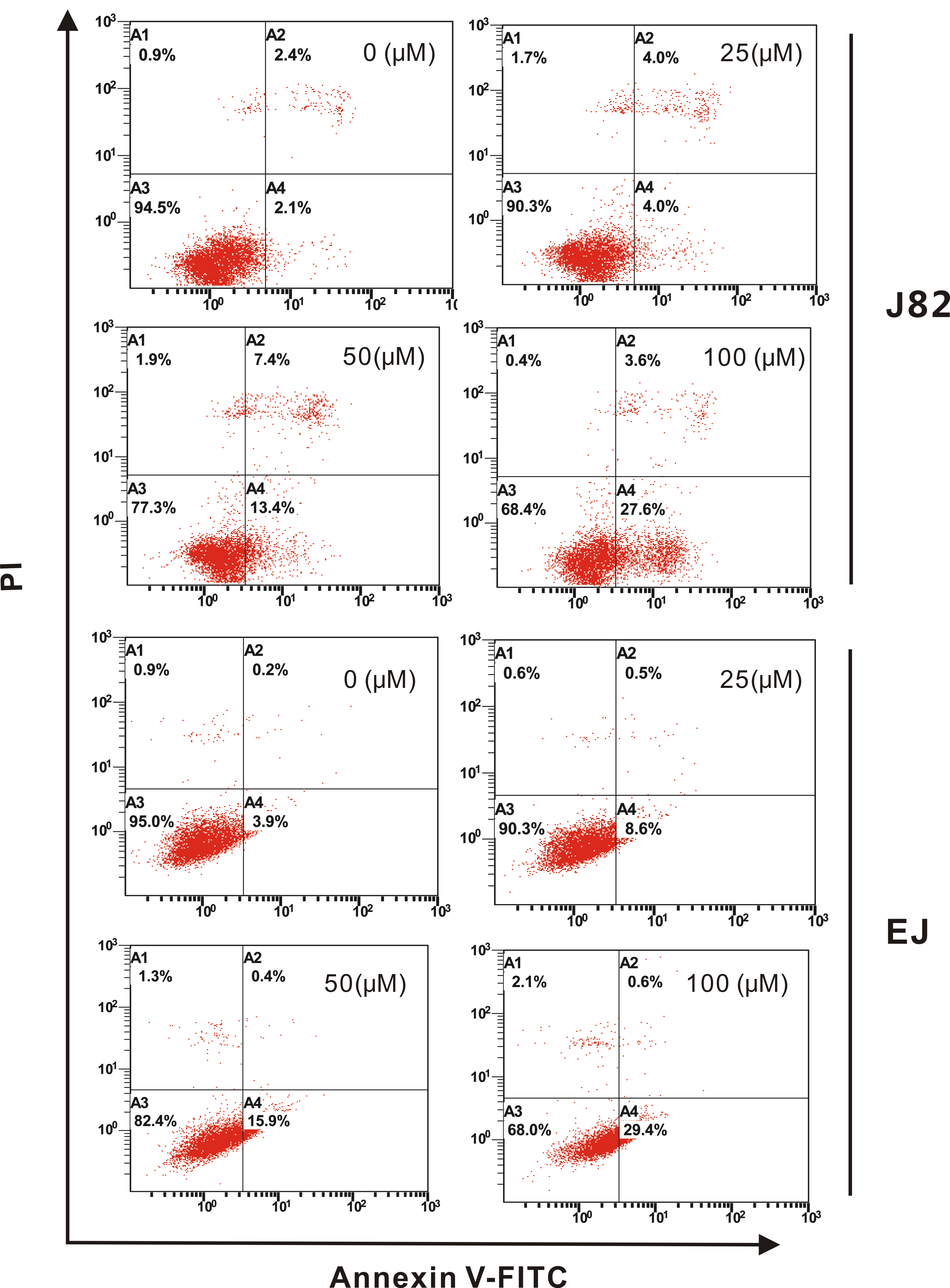

Supplement: S2 Fig — (TIF) [file pone.0161886.s002.TIF]

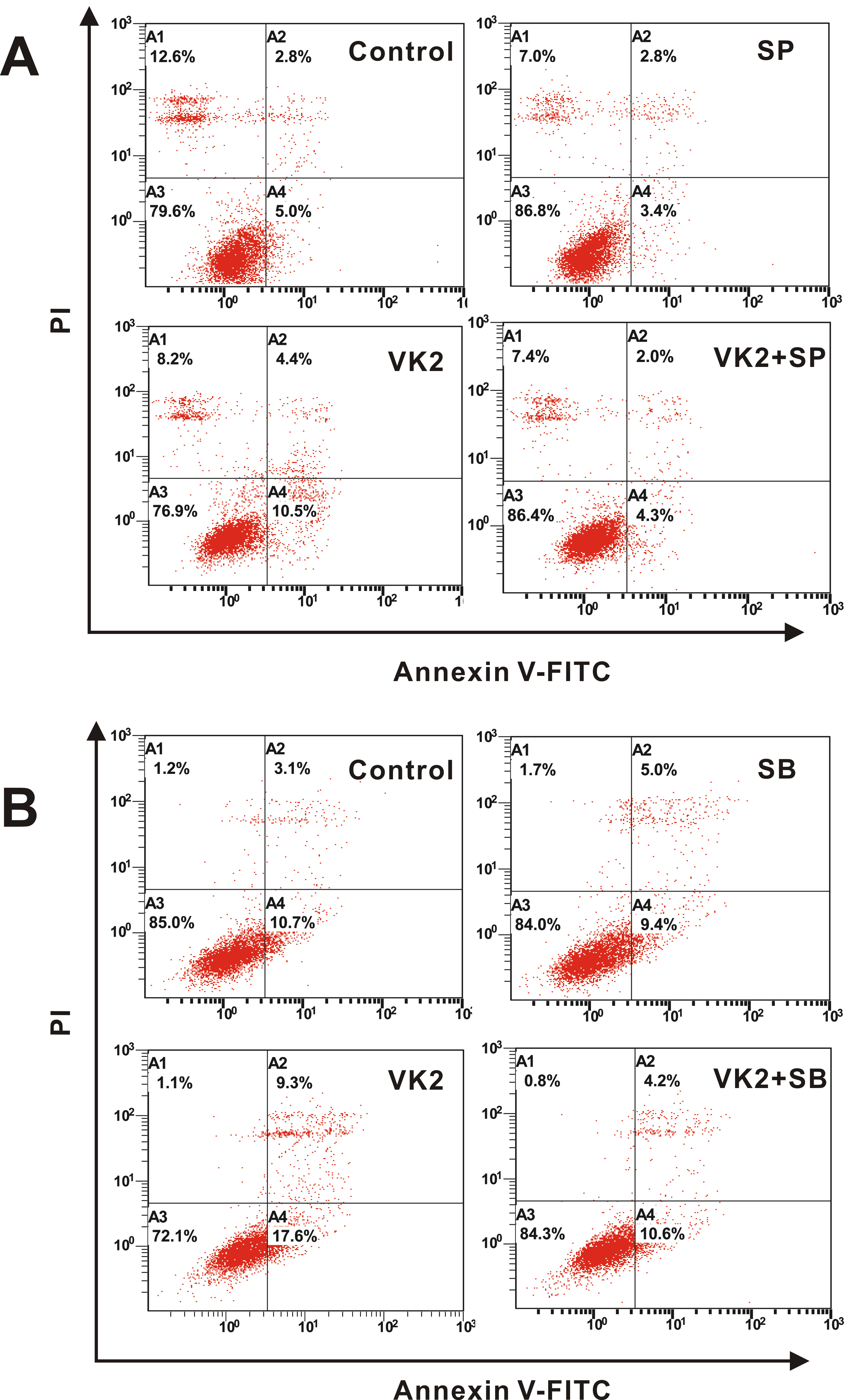

Supplement: S3 Fig — (TIF) [file pone.0161886.s003.TIF]

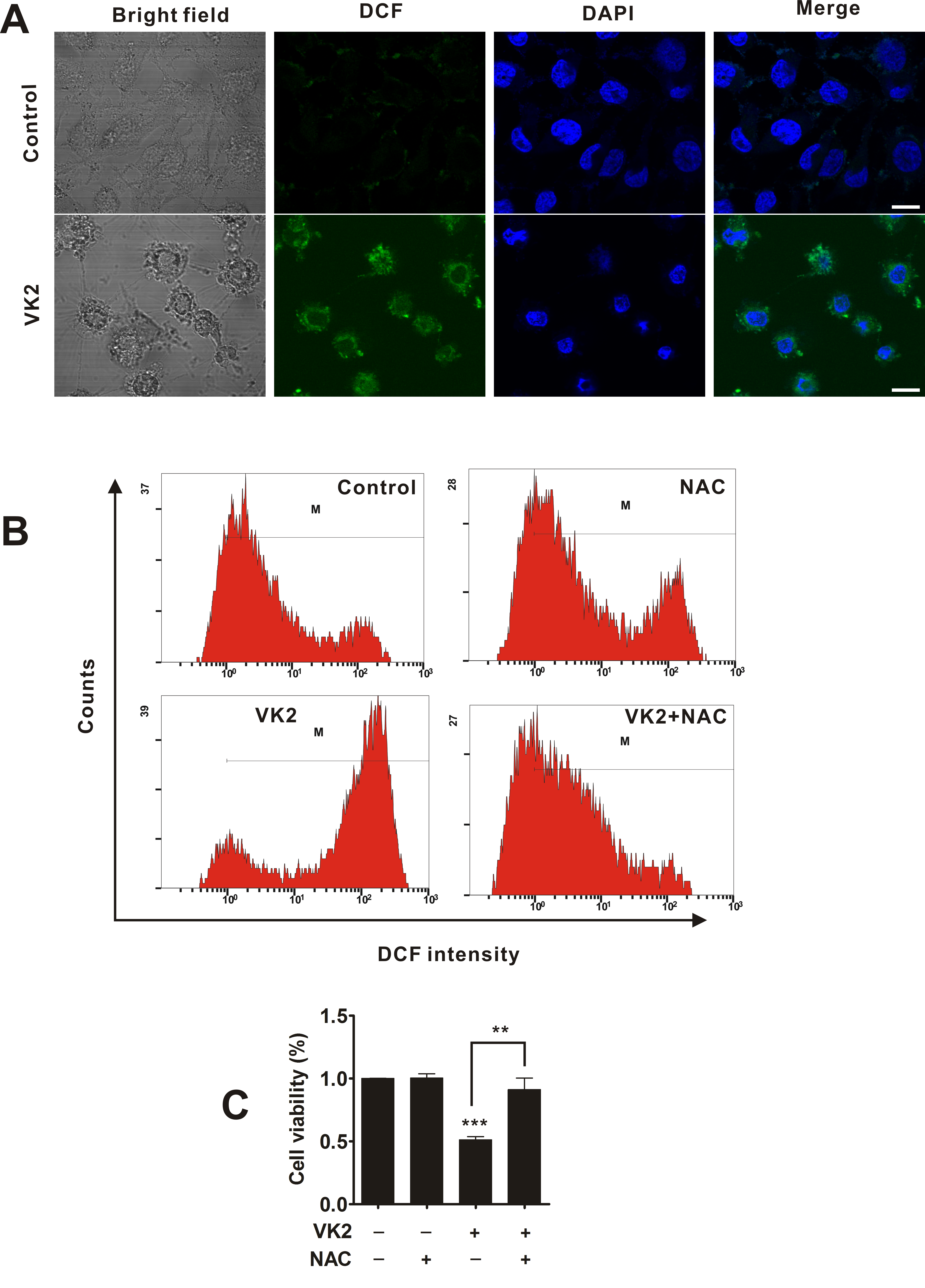

Supplement: S4 Fig — (TIF) [file pone.0161886.s004.TIF]
